# Deterministic and stochastic effects drive the gut microbial diversity in cucurbit-feeding fruit flies (Diptera, Tephritidae)

**DOI:** 10.1371/journal.pone.0313447

**Published:** 2025-01-24

**Authors:** Wouter Hendrycks, Nele Mullens, Jackline Bakengesa, Sija Kabota, Jenipher Tairo, Thierry Backeljau, Ramadhani Majubwa, Maulid Mwatawala, Marc De Meyer, Massimiliano Virgilio

**Affiliations:** 1 Department of Biology, Royal Museum for Central Africa (RMCA), Tervuren, Belgium; 2 Department of Biology, Evolutionary Ecology Group, University of Antwerp, Wilrijk, Belgium; 3 Department of Crop Science and Horticulture, Sokoine University of Agriculture, Morogoro, Tanzania; 4 Department of Biology, University of Dodoma, Dodoma, Tanzania; 5 Department of Biology, Royal Belgian Institute of Natural Sciences (RBINS), Brussels, Belgium; Bayero University Kano, NIGERIA

## Abstract

Insect diversity is closely linked to the evolution of phytophagy, with most phytophagous insects showing a strong degree of specialisation for specific host plants. Recent studies suggest that the insect gut microbiome might be crucial in facilitating the dietary (host plant) range. This requires the formation of stable insect-microbiome associations, but it remains largely unclear which processes govern the assembly of insect microbiomes. In this study, we investigated the role of deterministic and stochastic processes in shaping the assembly of the larval microbiome of three tephritid fruit fly species (*Dacus bivittatus*, *D*. *ciliatus*, *Zeugodacus cucurbitae*). We found that deterministic and stochastic processes play a considerable role in shaping the larval gut microbiome. We also identified 65 microbial ASVs (Amplicon sequence variants) that were associated with these flies, most belonging to the families Enterobacterales, Sphingobacterales, Pseudomonadales and Betaproteobacterales, and speculate about their relationship with cucurbit specialisation. Our data suggest that the larval gut microbiome assembly fits the “microbiome on a leash” model.

## Introduction

Phytophagous insects are highly diverse, both in species richness and their dietary habits/range [1,2]. Some of them are generalists that can attack a wide range of host plants from different plant families, while others specialise on one or a few closely related plant species [2]. This extensive dietary diversification is partly related to the rich communities of micro-organisms that insects harbour inside their guts [3]. These micro-organisms can participate in various functions relevant to the insect host, such as supplementation of nitrogen and vitamins, aiding in the breakdown of complex polysaccharides and the detoxification of dietary toxins [3–6]. For example, larvae of the olive fly *Bactrocera oleae* (Rossi, 1790) (Diptera, Tephritidae) depend on their symbiotic bacterium “Candidatus *Erwinia dacicola*” to feed on unripe olives that are rich in allelochemicals and lack nitrogen [4,5,7,8].

Microbes might play a pivotal role in explaining the dietary diversification of phytophagous insects by mediating host plant switching and host plant specialisation on chemically defended plants by aiding to overcome the chemical defences of these host plants, as proposed by the microbial facilitation hypothesis [3]. For example, in whiteflies, short-term adaptation to a less suitable host plant has been associated with changes in their gut microbiomes [9]. Dietary specialisations on particular host plant groups are related to specific microbiome profiles such as those found in insects feeding on cycads or on bamboo [10,11]. In aphids, different species feeding on the same host plant species show considerable overlap in their microbiome composition [12], and signatures of host plant specificity have been detected [13]. However, evidence of a role of the insect microbiome in host plant adaptation and specialization of the insect host is limited to a small number of case studies and it remains unclear to what extent and how it contributes to these processes.

For microbiomes to be involved in the host plant adaptation of the insect host, specific functional associations between the host insect and the microbiome must remain stable across generations. These stable associations might be generated by the host insect filtering out bacterial species sampled from the environment through host insect traits [3,14,15]. In some insects, specific structures in the gastrointestinal tract prevent the colonisation of the guts by specific bacteria [16]. Insect traits can determine the gut habitat and its available microbial niches in various ways, such as through the immune system [17]. In addition, maintained associations can arise through the vertical transmission of specific symbionts across generations [14,18,19]. However, the composition of insect microbiomes is not only determined by host filtering but is also strongly impacted by environmental factors such as diet and temperature [11,20,21]. For example, in bamboo-feeding insects, the abundance of some microbes is correlated to the concentrations of certain nutrients [11]. In tephritids, the host fruit considerably affects the larval microbiome [21–23].

Since insect-associated microbiomes can be regarded as meta-communities [24], the importance of host-associated processes in shaping the insect microbiome can be investigated through community assembly. Both deterministic processes and stochastic processes govern the assembly of ecological communities. Deterministic processes pertain to (a) environmental filtering (including host insect filtering) in which the environmental conditions determine microbiome community composition and (b) biological interactions between microbial species, such as competition and facilitation [25,26]. Bacterial community assemblies are also influenced by stochastic processes including e.g. ecological drift (fluctuations in species abundance due to birth, death, emigration and immigration), diversification and probabilistic dispersal [26]. How important deterministic and stochastic factors are in shaping the assembly of insect-associated microbiomes remains largely unexplored.

The few focused studies that have been conducted yielded dissimilar results, with some studies showing a critical role for host filtering [15], and, other studies indicating a largely role of stochastic factors [27,28].

True fruit flies (Tephritidae) are a diverse group of herbivorous flies which show varying degrees of host plant specialisation. About half of all tephritid species have frugivorous larvae that develop inside a wide variety of fruit species [29]. Associations with bacteria have long been known within Tephritidae and appear to play an important role in inducing fruit rot, nitrogen supplementation and overcoming host plant defences [4,5,14,30]. However, most studies on the tephritid microbiome have primarily focused on generalist fly species, especially from the genera *Bactrocera* and *Ceratitis*, and/or used laboratory populations, despite growing evidence that the microbiomes of laboratory colonies are not representative of those of natural populations [21,31]. Given that many tephritids are notorious agricultural pests [29], there is a clear need to understand better the relations between their gut microbiome and their dietary ecology.

In this study, we used 16S metagenomic amplicon sequencing to investigate the gut microbiomes of third instar larvae of three oligophagous (i.e. specialised in feeding on host plants belonging to the same family) tephritids: *Zeugodacus cucurbitae*, *Dacus ciliatus* and *Dacus bivittatus*. These species feed on Cucurbitaceae, a plant family known to produce allelochemicals (e.g. cucurbitacins), which are toxic to insects [32,33]. These flies also show a high degree of overlap in their host fruit range [34], allowing us to control for confounding effects due to interspecific dietary ecology. The primary objective of this study was to investigate the role of deterministic and stochastic processes in shaping the larval microbiome of fruit flies. In this respect, we tested whether (1) interspecific phylogenetic relatedness and (2) intraspecific phylogenetic and taxonomic turnover of the microbial communities differ significantly from stochasticity and (3) whether larval microbial assemblies fit a neutral model. In this context, we quantified signals of positive selection in bacterial taxa shared across the three target flies. Lastly, we estimated the relative contribution of insect filtering and host plants in shaping the larval microbiome of these cucurbit-feeding flies.

## Material and methods

### Field sampling

We studied larvae of three cucurbit feeding tephritid species: *Zeugodacus cucurbitae* (Coquillett, 1899), *Dacus ciliatus* Loew, 1862 and *Dacus bivittatus* (Bigot, 1858). Third instar larvae from these species were collected at 20 experimental sites of approximately one hectare each in Morogoro in Eastern Central Tanzania. Ten sites were located at the plains of the base of the Uluguru mountains (~500 m elevation) and 10 sites were located in the Uluguru mountains (~1000 m elevation). From each site, fruits from three different cucurbit crops were collected: cucumber (*Cucumis sativus* Linnaeus), watermelon (*Citrullus lanatus* (Thunb.) Matsum & Nakai) and squash (*Cucurbita pepo* Linnaeus). Collected fruits were brought to the horticultural unit at the Sokoine University of Agriculture (SUA) in Morogoro and were placed in plastic boxes that were covered with a fine mesh cloth and that contained a substrate of sand. These boxes were frequently checked for the presence of third instar larvae, either in the sand or inside the fruits by dissection. If present, third instar larvae were immediately collected and rinsed with phosphate buffered saline solution (PBS). Larvae were killed by placing them in individual tubes with 98% ethanol and stored at -20°C in 98% ethanol for microbiome profiling. From each site, we collected 0–20 third instar larvae from each host fruit species. The Sokoine University of Agriculture (SUA) approved and regulated the field site access in collaboration with the local authorities. Because the Nagoya protocol on Access and Benefit sharing is not implemented in Tanzania, Mutually Agreed Terms (MATs) established between SUA and RMCA on the use of genetic resources regulates the intellectual and physical property of the samples collected in this study. This document is inspired and adheres to the principles of the Nagoya Protocol and is provided in S1 File. As pest insects do not fall under the European Directive 2010/63/EU on the protection of animals used for scientific purposes, we did not need to have approval for this study by an animal research ethics committee.

### Laboratory procedures

As De Cock et al. [35] did not detect any significant differences between whole body and gut microbiome profiles, we used whole body DNA extracts from third instar larvae for both larval identification and microbiome profiling. Identification of the larval species (which is problematic through morphological characters, see Pieterse et al. [36]) was done through DNA barcoding using mitochondrial COI reference libraries for African tephritids [37]. Laboratory procedures followed De Cock et al. [38] using the DNeasy Blood and Tissue kit (Qiagen Inc, Hilden, Germany) unless indicated otherwise. Prior to lysis, larvae were cut in half to ensure penetration of the products into the larval tissues. The universal COI primers LCO1490 and HCO2198 were used for DNA barcoding all samples. Sequencing was done by Macrogen (https://www.macrogen.com). Species identification was done by blasting sequences against the BOLD database (https://www.boldsystems.org/).

Microbiome characterization was done through DNA metabarcoding of the V3-V4 regions of the 16S rRNA gene as described in Hendrycks et al. [21]. Read quality was verified with FastQC [39]. The DADA2 pipeline [40] was used to remove primers and truncate reads (to a final length of 280 bp for forward reads and 220 bp for reverse reads resulting in ca. 36 bp overlap) and to fit the parametric error model for the identification and filtering of sequencing errors. Filtering was based on maxEE = 1,1 in DADA2. The first 300 million bases were used in error estimation for constructing the error model. Before pairing forward and reverse reads and filtering out chimeras, unique amplicon sequence variants (ASVs; 100% unique sequence identity) were extracted using the Bayesian classifier method of DADA2. We chose to use ASVs instead of OTUs as ASVs are replicable unlike de novo OTUs [41] The Silva v.132 database was used for the taxonomic classification of ASVs (percentage of identity = 97% similarity, p‐min‐ consensus = 0.51). Contamination sequences were removed using a negative control with the R package MicroDecon v1.0.2 [42]. We also removed mitochondrial and chloroplast sequences prior to statistical analysis.

Prior to statistical analysis, we applied a 0.1% relative abundance filter as in Reitmeier et al. [43] to remove spurious sequences. Statistical analyses were performed in R unless stated otherwise. Permutational analysis of variance (PERMANOVA) was conducted with PRIMER-E v7.0.21 [44] using 9,999 permutations of residuals under the full model. The entire bioinformatic and statistical pipeline can be found at Zenodo at https://doi.org/10.5281/zenodo.11362674. Raw sequencing data has been deposited at the European Nucleotide Archive (ENA) with accession number PRJEB77429.

### Microbiome diversity analysis

An overview of the experimental design can be found in S1 Table. Due to the limited number *D*. *ciliatus* larvae found on watermelon and lack of *D*. *ciliatus* larvae on cucumber, a subset of the experimental design was used for specific analyses that required a balanced design (S1 Table, part B). As larvae of the different species were patchily found across the different sites and altitudes, it was not possible to create a balanced subset design with site and altitude as factors. As a consequence, we excluded the effect of site and altitude in the subsequent statistical analysis.

Prior to calculating β diversities, counts were normalized by transforming them into proportions to represent community structure [45,46]. Generalized UniFrac distances using the d5 matrix and unweighted UniFrac distances were calculated as β diversity metrics [47]. As UniFrac distances take into account phylogenetic relationships, a phylogenetic tree needs to be constructed. Multiple alignment of the bacterial 16S sequences was done with the DECIPHER package v2.14.0 [48] prior to tree building. The program Fasttree v2.1.11 was used to construct a midpoint‐rooted maximum likelihood tree of the bacterial relationships using a general time‐reversible substitution model [49].

Differences in microbiome β diversity were tested on a balanced subset of samples (S1 Table, part B) to test for significant differences in microbial α diversity between larvae from different fly species and/or from different host plants, with fly species (*D*. *ciliatus*, *D*. *bivittatus* and *Z*. *cucurbitae*) and host plant (watermelon and squash) as a fixed orthogonal factor using a two‐way PERMANOVA [50] with fly species and host plant as fixed orthogonal factors with sum of squares type III. The false discovery rate (FDR) correction [51] with experiment‐wise p < 0.05 was used to correct for multiple testing. PERMANOVA was also used to estimate the components of variance explained by each factor. As *D*. *ciliatus* was not found on cucumber, comparisons between all three host plants (cucumber, watermelon and squash was restricted to *Z*. *cucurbitae* and *D*. *bivittatus*). Differences between larvae feeding on different host plants were visualized with a principal coordinate analysis (PCoA) using the ggplot2 package v3.3 [52].

### Null model analysis

To determine the relative influence of deterministic vs stochastic processes in shaping the microbiome communities of flies, we used a consensus approach consisting of two different approaches: a null model approach and a neutral model approach.

To examine whether larval microbiomes are assembled by deterministic processes we looked at patterns of phylogenetic relatedness between microbial species within the same community. To measure the extent of phylogenetic relatedness in a community, we calculated the MNTD (mean nearest taxon distance) which calculates the average phylogenetic distance between all taxa and their closest relative within individual larval communities using the picante package v1.8.2 [53]. We choose this index as it only looks at the phylogenetic relatedness between closely related species because phylogenetic signals of ecology are expected to occur in bacteria only across short phylogenetic distances due to their rapid evolution [54]. We chose to use an abundance weighted MNTD as incorporating abundance information improves the power to detect assembly processes [55]. In order to detect patterns of phylogenetic relatedness higher or lower than expected, we deployed null models to generate a distribution of what the expected phylogenetic relatedness of the community would be under randomized conditions and subsequently compare the observed MNTD to the expected MNTD by calculating the standardized effect sizes (SES.MNTD). We chose to deploy a consensus approach using 2 different null models to randomize the data. The taxa.labels null model randomizes the phylogenetic relatedness of community members. As this test can be quite liberal [56,57], we also employed the highly conservative independent swap algorithm which fixes both sample species richness and species occurrence rates [56–58]. We applied each null model using 1000 permutations for each individual community. To account for larvae feeding on different host plants might be exposed to different microbial source pools, SES.MNTD values were calculated for larvae within each host plant treatment separately. Across communities, a mean SES.MNTD significantly higher than zero indicates phylogenetic repulsion while a mean SES.MNTD significantly lower than zero indicates clustering. To test whether observed ses.MNTD values were different from 0, two-tailed t tests were employed.

We also investigated community assembly by looking whether there were patterns in turnover between larval microbiome communities, focusing on both phylogenetic turnover and taxonomic turnover. Taxonomic turnover is the degree of change that occurs in species composition between different ecological communities while phylogenetic turnover can be regarded as the extent of phylogenetic relatedness that occurs between the set of microbial species in community A and the set of microbial species in community B. If deterministic processes largely determine the differences in species composition between different microbiome communities, we would expect higher or lower phylogenetic relatedness between species from the different communities than when species composition of different communities is governed by purely neutral processes [58]. To measure phylogenetic turnover we used the abundance weighted βMNTD (Beta Mean Nearest Taxon Distance) which is the between communities analogue for the MNTD index. As with the MNTD index, a null distribution of the expected phylogenetic turnover was generated by using a tip-shuffle null model with 1000 permutations for each community comparison (between larvae of the same fly species within each host plant treatment separately) and compared to the observed phylogenetic turnover by calculating the standardized effect sizes (SES.βMNTD). Across comparisons, a mean SES.βMNTD significantly higher than zero indicates phylogenetic repulsion while a mean SES.βMNTD significantly lower than zero indicates clustering [59]. To test whether observed ses.βMNTD values were different from 0, two-tailed t tests were employed. Taxonomic turnover was measured using the modified Raup-Cricke index that also takes into account abundances [59]. For a single comparison, values > |0.95| indicates that turnover is greater or smaller than expected which can be indicative of either deterministic or dispersal processes [59]. As with phylogenetic turnover, we used two-tailed t tests to test whether observed Raup-Cricke values were different from 0.

### Neutral model analysis

While null models can be a powerful tool to detect non-random or non-neutral community assembly patterns, they are based on heuristic randomization schemes that exclude clear biological mechanisms. As a consequence, rejection of the null hypothesis by these null models might simply reflect an inability of the null model to realistically reflect the effect of neutral processes on community assembly [58]. Moreover, null model analysis on large ecological datasets such as microbiomics data might be more prone to type 1 errors [60]. To validate the results obtained from the null model analysis, we also estimated the importance of stochastic processes in community assembly by fitting our data to a mechanistic neutral model. We chose the Sloan neutral model as it has been specifically designed for the analysis of microbiomics data [24]. This model predicts the relation between the prevalence of microbial species across communities and their mean relative abundance across these communities. As such, the Sloan neutral model predicts that more abundant microbial species will also be more prevalent, while rare microbial species will be more subjected to ecological drift [24]. Using this relationship, the model can be used to estimate what percentage of microbial species in a dataset have an abundance-prevalence relationship that fits the neutral model, i.e. that can be used as a measure of the influence of stochastic processes on community assembly. Microbial species that deviate from this expectation may be indicative of deterministic processes, such that higher prevalences than expected indicate positive selection by the insect host, while lower prevalences than expected indicate negative selection, competition and/or dispersal limitation [61,62]. The model is fit to the observed ASVs prevalence and abundance by a single free parameter, the migration rate m, which represent the probability that an individual is replaced by immigration rather than local demographic processes [24]. A crucial assumption of this neutral model is that there is no speciation in the community. We do not regard this as a problem as we sampled third instar larvae that were at most a few weeks old, making microbial speciation unlikely.

For the model fitting we used the model fitting approach of Burns et al. [61] using the snm function in the iCAMP R package v1.6.4 [63]. The goodness of fit of the data to the neutral model was assessed using the root mean square deviation (RMSE). To determine whether incorporating stochastic effects improved the fit outside of random sampling, we also compared to the fit to a binomial model. Using this neutral model, we could also identify core (stable associates) bacterial genera and ASVs that might be associated with cucurbit feeding by looking at which microbial species are more prevalent than expected as these indicate selection signatures exerted by the host insect [61].

### Ethics statement

Benefits from this research accrue from the sharing of our data, analysis and results on public platforms. Materials/samples obtained and used in this study comply with a Mutually Agreed Terms arrangement with the Tanzanian partners in accordance with Nagoya.

## Results

The MiSeq Illumina run resulted in more than 25.1 × 10^6^ reads (average number of reads / sample = 102,187; SD = 43,451). Following filtering, demultiplexing, contaminant removal and abundance filtering, 1 × 10^7^, belonging to 2085 unique ASVs, were retained.

### Microbiome diversity analysis

Differences in β diversity were revealed by PERMANOVA on the Generalized and Unweighted Unifrac distances. There was a significant interaction between host plant diets and fly species for both distances (S2 Table). Pairwise comparisons showed that within watermelon, there were no differences between larvae of *Z*. *cucurbitae* and *D*. *ciliatus* for both Unifrac distances. All other pairwise comparisons were significant (S2 Table). For the Unweighted Unifrac distances, our results showed that fly species explained 15.5% of the variation, host plant 11.8% and their interaction 12.4%. For the Generalized Unifrac distances, 19.8% of variation was explained by fly species, 14.2% by host plant and 11% by their interaction. PCoA of presence/absence data (Unweighted Unifracs, 31.2% variation explained) and weighted abundances (Generalized Unifracs, 36.8% variation explained) showed a clear separation between the larval microbiomes of *Z*. *cucurbitae* and *D*. *bivittatus* (S1 Fig).

### Null model analysis

**Fig 1:**
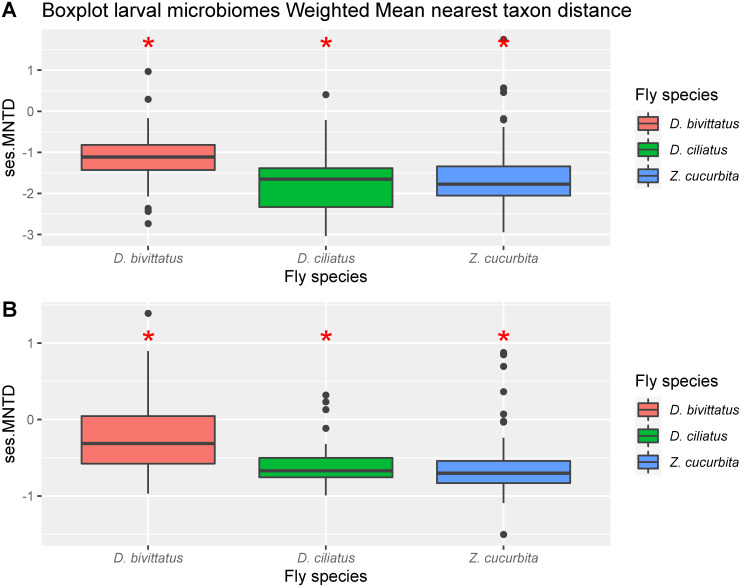
Standardized effect size Mean Nearest Taxon Distances (ses.MNTD) for microbial communities of third instar larvae of different fly species (*D*. *ciliatus*, *D*. *bivittatus*, *Z*. *cucurbitae*) as calculated from Abundance weighted metrics. Upper panel are estimated using the taxa.labels null model while lower panel are estimated with the independent swap model. * indicate which treatments differed significantly from 0.

In the analysis of the phylogenetic community structure, all three cucurbit feeders showed significantly low mean MNTD effect sizes for all three species for both null models (Table 1 and Fig 1), indicating a considerable level of phylogenetic attraction.

**Table 1:** Two sided t-test on A) Weighted ses.MNTD with taxa.labels null model and B) Weighted ses.MNTD with independent swap null model calculated from third instar larvae.

**A) Weighted NTI**	**Mean**	**df**	**t-value**	**P-value**
*D*. *bivittatus*	-1.144	47	-12.142	<0.001***
*D*. *ciliatus*	-1.744	25	-10.696	<0.001***
*Z*. *cucurbitae*	-1.510	47	-11.169	<0.001***
**B) Weighted NTI**	**Mean**	**df**	**t-value**	**P-value**
*D*. *bivittatus*	-0.199	47	-2.7505	<0.008**
*D*. *ciliatus*	-0.570	25	-8203	<0.001***
*Z*. *cucurbitae*	0.556	47	7.468	<0.001***

Calculated for different fly species (*Dacus ciliatus*, *D*. *bivittatus*, *Zeugodacus cucurbitae*) feeding on different host plants (*Cucumis sativus*, *Citrullus lanatus*, *Cucurbita pepo*). n.s.: Not significant;

*: P < 0.05,

**: P < 0.01,

***: P < 0.001.

**Fig 2:**
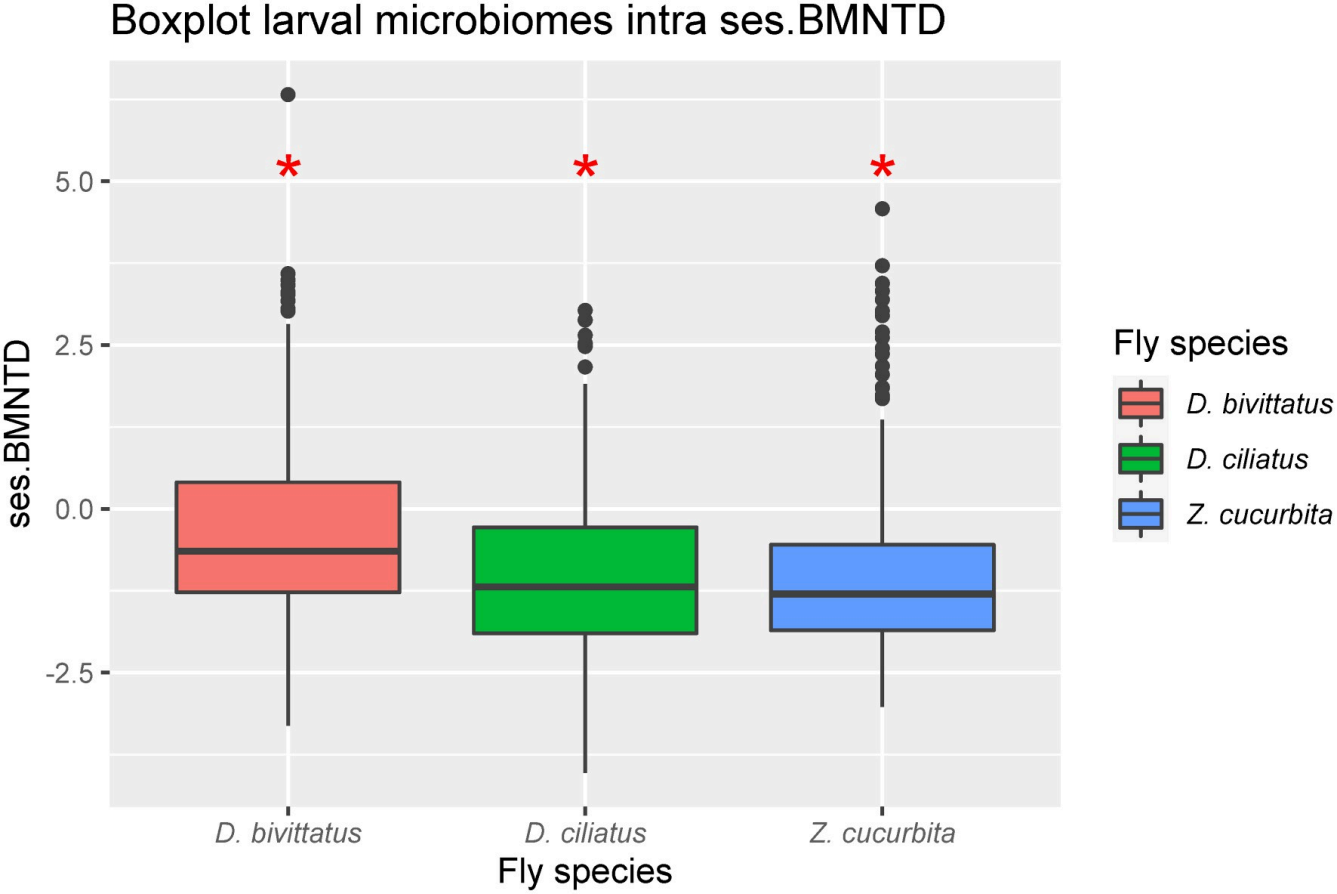
Intraspecific abundance weighted Standardized effect size β Mean Nearest Taxon Distances (ses.MNTD) for microbiomes. Calculated for third instar larvae of different fly species (*D*. *ciliatus*, *D*. *bivittatus*, *Z*. *cucurbitae*). * indicate which treatments differed significantly from 0.

**Fig 3:**
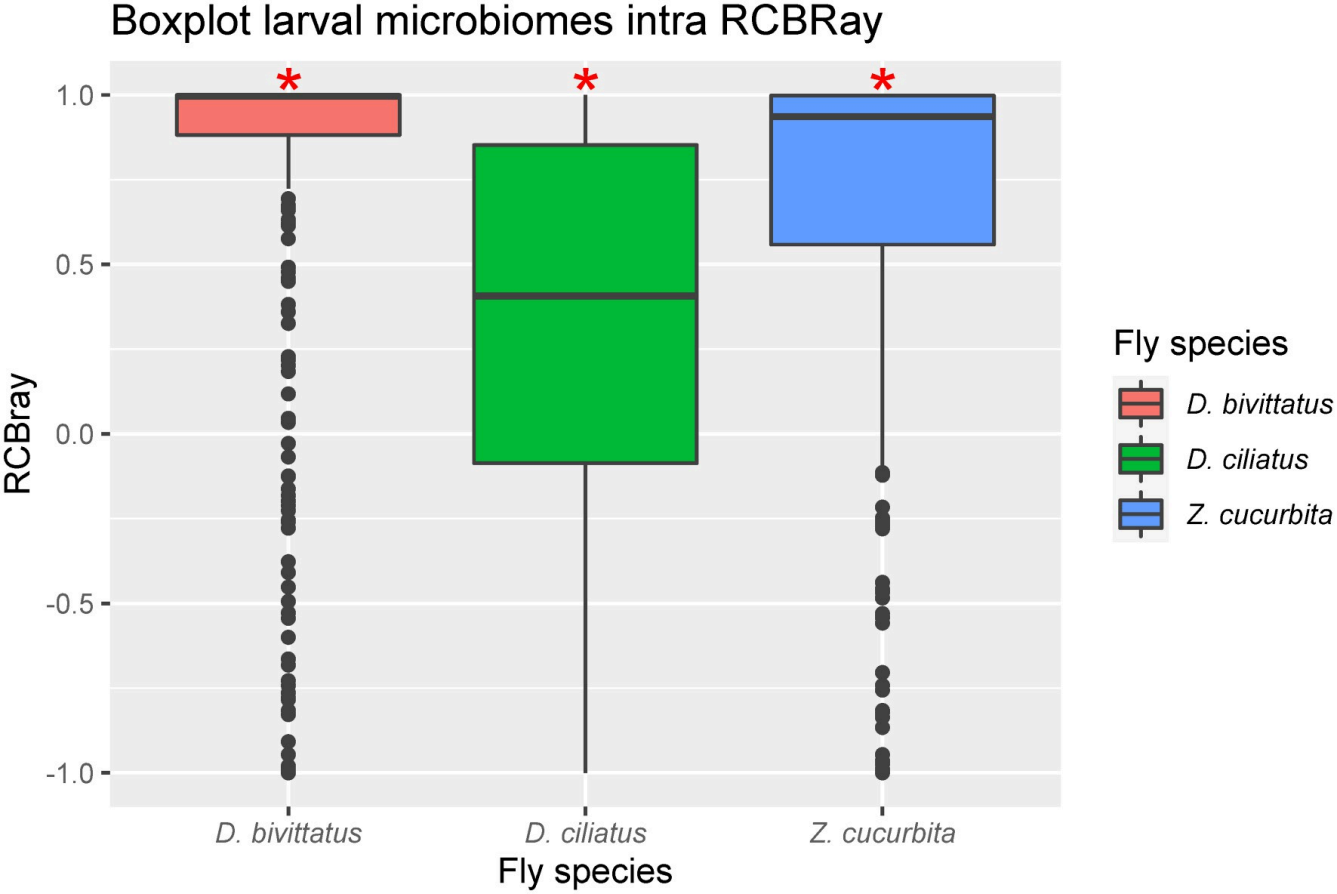
Intraspecific abundance weighted Raup-Cricke Bray distances (RCBray) for microbiomes. Calculated for third instar larvae of different fly species (*D*. *ciliatus*, *D*. *bivittatus*, *Z*. *cucurbitae*). * indicate which treatments differed significantly from 0.

All three species also showed significantly low mean βMNTD effect sizes and significantly high RCBray values indicating low phylogenetic turnover despite high taxonomic turnover between individual larval samples of the same fly species (Table 2, Figs 2 and 3).

**Table 2:** Two sided t-test on A) Weighted ses.βMNTD with taxa.labels null model, B) the modified Raup-Cricke Bray index.

**A) Weighted βNTI**	**Mean**	**df**	**t-value**	**P-value**
*D*. *bivittatus*	-0.366	359	-5.063	<0.001***
*D*. *ciliatus*	-0.943	164	-8.511	<0.001***
*Z*. *cucurbitae*	-0.987	359	-14.842	<0.001***
**B) Modified RCBray**	**Mean**	**df**	**t-value**	**P-value**
*D*. *bivittatus*	0.723	359	23.654	<0.001***
*D*. *ciliatus*	0.293	164	5.937	<0.001***
*Z*. *cucurbitae*	0.654	359	23.060	<0.001***

Calculated from third instar larvae from different fly species (*Dacus ciliatus*, *D*. *bivittatus*, *Zeugodacus cucurbitae*) feeding on different host plants (*Cucumis sativus*, *Citrullus lanatus*, *Cucurbita pepo*). n.s.: Not significant;

*: P < 0.05,

**: P < 0.01,

***: P < 0.001.

### Neutral model analysis

**Fig 4:**
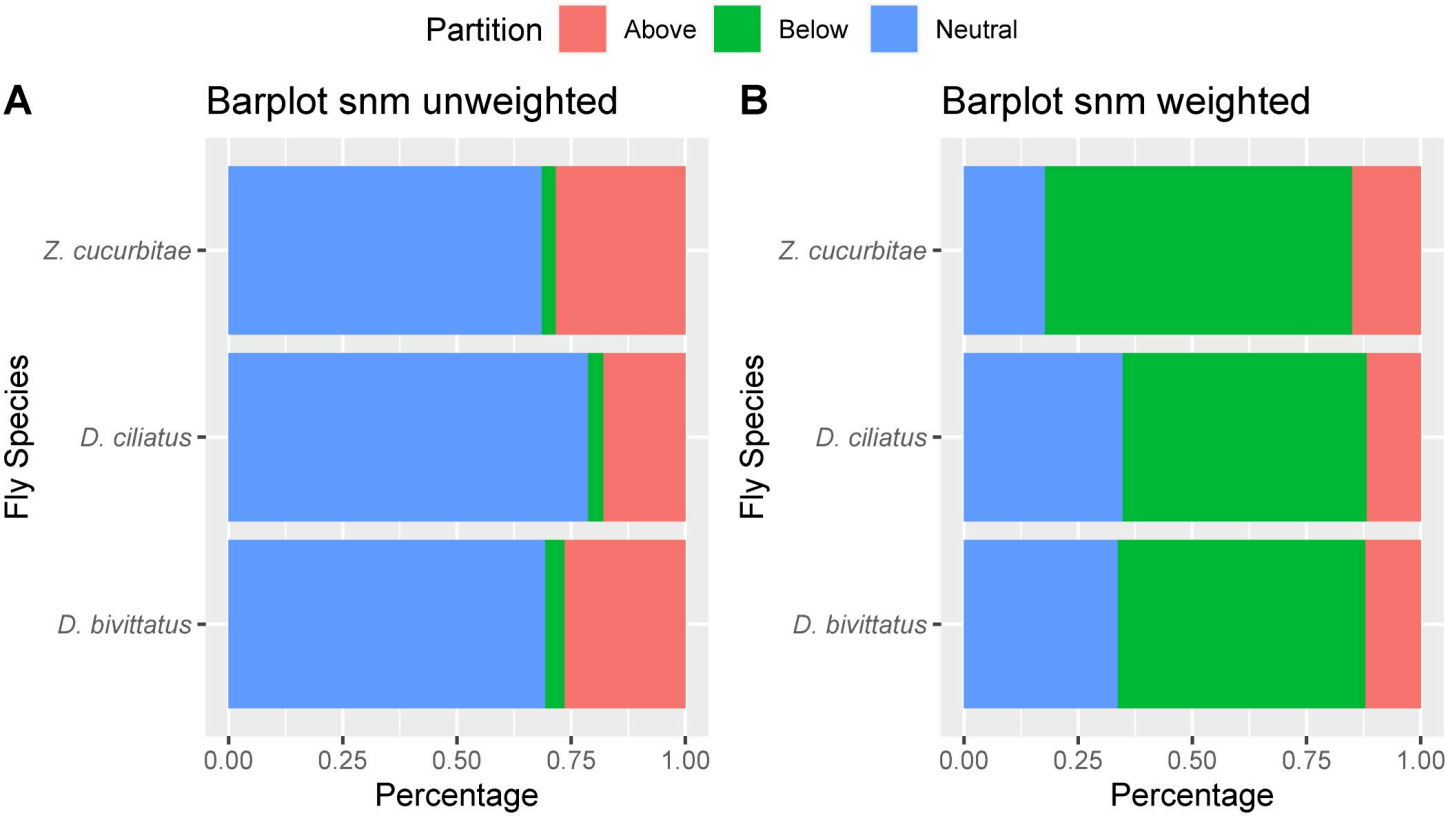
Barplots showing A) the relative proportions of bacterial taxa that fit either the neutral model, are more prevalent than expected (Above) or less prevalent than expected (Below). B) the relative proportions of reads that belong to bacterial taxa that fit the neutral model, are more prevalent than expected (Above) or less prevalent than expected (Below).

Neutral model analysis showed a poor fit to the model for the three fly species with for *Z*. *cucurbitae* (RMSE = 0.116), for *D*. *bivittatus* (RMSE = 0.128) and for *D*. *ciliatus* (RMSE = 0.138), indicating an important role of deterministic processes in the larval microbiome assembly. For all fly species, the neutral model outperformed the binomial model, indicating a role for stochastic processes in community assembly (*D*. *bivittatus*: RMSE = 0.72; *D*. *ciliatus*: RMSE = 0.71; *Z*. *cucurbitae*: RMSE = 0.701). Unweighted partitioning of the neutral model showed that 69–79% of all bacterial species have a prevalence-abundance ratio that fits the neutral model (i.e. they are as prevalent as expected for their mean abundance under the assumption of neutral assembly) indicating a strong effect of stochastic processes on microbiome assembly (Fig 4 and S3 Table). Of all bacterial species, 18–28% were overrepresented in the unweighted partitioning (higher prevalence than expected for their mean abundance) while 3–4% of bacterial species were underrepresented (less prevalent than expected for their mean abundance). When taking into account the relative abundance of the bacterial species, weighted partitioning of the neutral model found that only 18–35% of the relative abundance was explained by the neutral expectation (Fig 4 and S3 Table). The portion of underrepresented bacterial species explained 53–67% of the relative abundance while 12–15% of the relative abundance was attributed to the overrepresented bacterial species, indicating a strong effect of deterministic processes on relative abundance. The neutral model analysis found low migration rates of m = 0.0002–0.0007, suggesting strong dispersal limitation.

**Fig 5:**
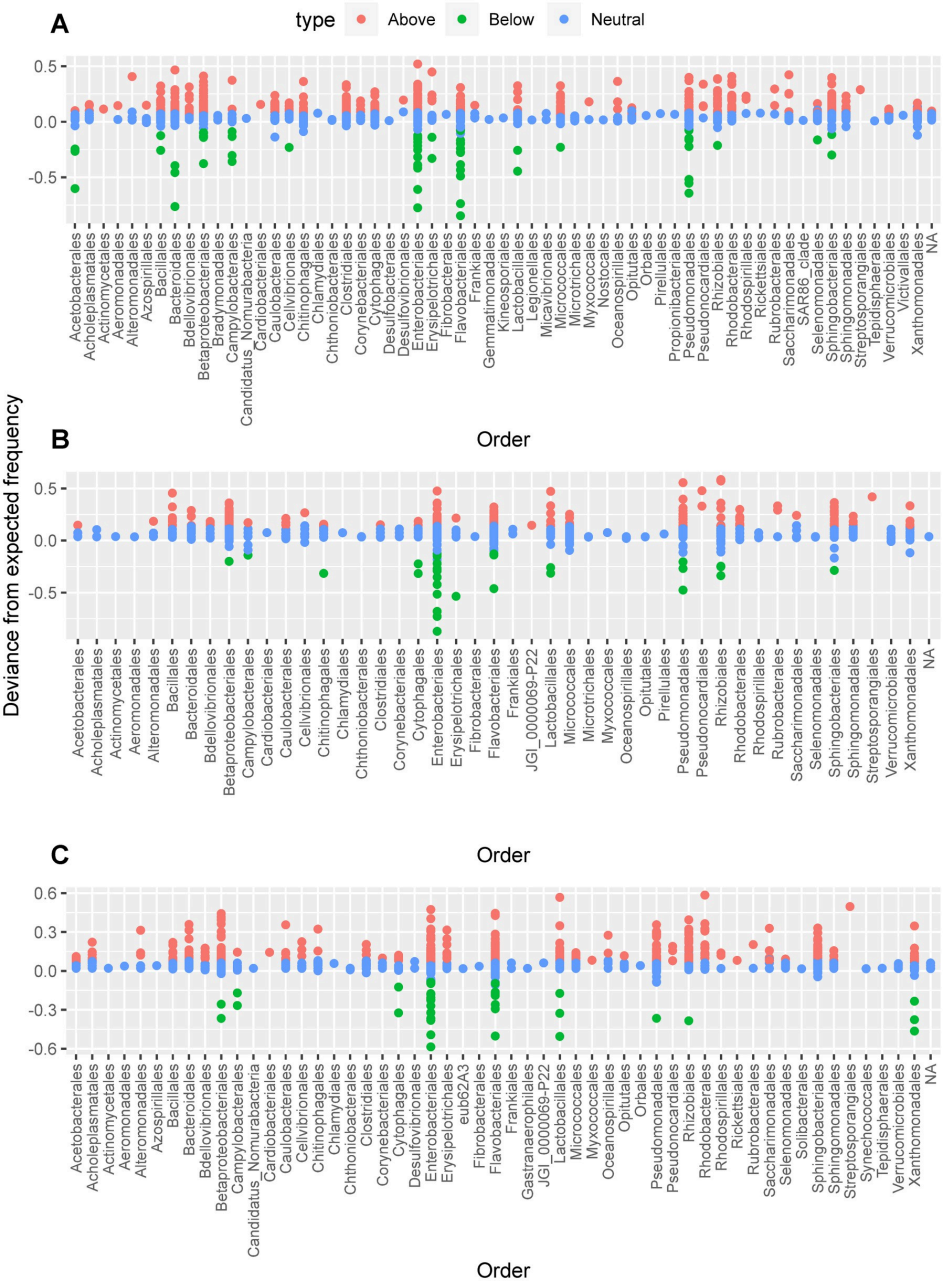
Taxonomic distributions of neutral, over- (Higher prevalence than expected) and underrepresented (Lower prevalence than expected) bacterial taxa for every cucurbit feeder with respect to the neutral model fit. Above: *D*. *bivittatus*; Middle: *D*. *ciliatus*; Bottom: *Z*. *cucurbitae*.

We also employed the neutral model of Sloan (Sloan et al., 2007) to identify bacterial ASVs that showed signatures of positive selection (higher prevalence than expected). Neutral model analysis indicated that 65 ASVs showed signatures of positive selection among all the tested cucurbit feeders (Fig 5 and S4 Table). These belonged to 20 different orders, with most ASVs (33 out of 65) confined to Betaproteobacterales, Enterobacterales, Pseudomonadales and Sphingobacteriales. Only 4 ASVs showed signatures of negative selection (lower prevalence than expected) among all the tested cucurbit feeders. Out of all ASVs with negative selection signatures, 31.25% belonged to the Enterobacterales.

## Discussion

### The role of deterministic vs stochastic factors

Theory predicts that deterministic processes cause species in an ecological community to be more or less closely related than expected under stochastic conditions due to phylogenetic conservatism. For example, environmental filtering selects species with similar ecological traits causing phylogenetic relatedness, while negative biotic interactions such as competition promotes phylogenetic repulsion [25]. The present results show that the microbiomic assemblies of *Dacus bivittatus*, *D*. *ciliatus* and *Zeugodacus cucurbitae* are subjected to deterministic effects and suggest a role for host filtering. This is based on multiple lines of evidence: 1) larval microbial β diversity significantly differs between fruit fly species, even when feeding on the same host plant. Fly species identity also explained more variation than host plant. This suggests that different fly species filter different cohorts of bacteria. 2) The larval microbiome exhibits a considerable degree of phylogenetic attraction, particularly in *Z*. *cucurbitae* and *D*. *ciliatus*. This pattern is inconsistent with the predominant role of dispersal limitation, which promotes phylogenetic repulsion in both α and β phylogenetic community structures, [64]. 3) Our analyses show that a consistent part of all bacterial species (23–31%) did not fit the neutral model, suggesting that deterministic processes have a relevant impact on the microbial community. A large proportion (18–28%) of all bacterial species show higher prevalence than expected, suggesting a selection of these species by the larval host. 4) Abundance weighting reduced the fit to the neutral model (which then accounted for only 18–35% of microbiome relative abundance), further indicating the importance of deterministic effects on bacterial species abundances.

Our results also show that variability in microbiome composition between larvae of the same fly species corresponds to a high degree of taxonomic turnover, as indicated by the high RCbray values. This, however is not accompanied by a high phylogenetic turnover, suggesting that intraspecific differences in microbiome composition are mainly driven by substitutions of closely related bacterial taxa. This indicates that the microbiome variability is limited by phylogenetic boundaries imposed by deterministic processes. High and phylogenetically constrained variability is compatible with bacterial functional redundancy as it has been described in other microbial communities, including in arthropods [65–67] and microbial traits tend to show phylogenetic conservatism among close relatives [68]. These results suggest that the microbiome of these flies may form stable functional associations regardless of their highly heterogeneous taxon diversity. Therefore, it might be of interest for future studies to focus not only on the identification of core bacterial taxa but also a functional core microbiome *sensu* Risely [69] as important associations might be missed by solely focusing on bacterial taxa.

Interestingly enough, the neutral model analysis found a very low migration rate (m = 0.0002–0.0007), indicating strong dispersal limitation. The migration rates obtained in this study are several magnitudes lower than those observed in human gut microbiomes, which are known to be dispersal-limited [70]. Dispersal limitation is also consistent with the observed microbiome variability as we sampled larvae from different fruits and localities. Given that larvae develop inside the fruit, larvae can only obtain symbionts through vertical transmission and from the tissue of the individual fruit. It is known that bacteria with heterogeneous distributions colonise both the fruit skin and the fruit pulp [71,72]. As the fruit microbiome might influence the larval microbiome [21,22,31], substantial differences in microbial exposure might occur between individual larvae at very small spatial scales. This interpretation is supported by several studies showing high spatial variability in tephritid microbial patterns [21,22,31]. While vertical transmission is a relevant process occurring in several tephritids, including *Z*. *cucurbitae* [14,18,19], the low microbial dispersal observed in this study indicates that local environmental sampling might be more important for symbiont acquisition.

Overall, our results indicate that the larval microbiome is shaped by the interaction of deterministic processes such as host filtering, dispersal limitation and ecological drift and that these processes operate within the phylogenetic boundaries proposed by the *microbiome on a leash model* [73]. There are a variety of mechanisms by which host filtering could occur. Larvae could selectively modify their behaviour to specifically enhance or avoid the acquisition of specific bacteria as has been shown in *Ceratitis capitata* (Wiedemann, 1824) [74]. Larvae could also affect the establishment of microbes by modulation through their immune system, as it occurs in both *Drosophila* and *C*. *capitata*, where antimicrobial peptides on the egg surface selectively reduce specific microbial taxa [75,76]. Similarly, in *B*. *dorsalis*, the BDnub gene modulates the production of antimicrobial peptides, which can change gut microbiome composition [17].

The neutral model analysis showed that most of the relative abundance of the microbiome community is explained by a very small number of underrepresented bacterial species. A lower prevalence than expected by chance and suggests the occurrence of negative selection by the host insect as has been shown to occur for pathogenic bacteria in zebrafish [61]. Yet, other factors, including poor colonization capacity priority effects (i.e., bacterial species reaching high abundances only as early colonisers) might also promote low bacterial prevalence [26,61]. The neutral model analysis also showed that 18–28% of bacterial species were overrepresented. However, these species explained only 12–15% of the relative abundance, suggesting that overrepresented bacterial species tend to be less abundant. A pattern of overrepresentation can arise when a bacterial species plays a crucial role in the host insect biology [60]. For example, in mice, several species of the Ruminococcaceae are overrepresented, and these bacteria play an essential role in metabolising plant polysaccharides [61,77]. Moreover, rare bacteria often play crucial roles in community functioning [78,79], and rare bacterial species might mediate dietary shifts in tephritids [21]. However, overrepresentation can also arise when a bacterial species is a good coloniser, regardless if it experiences strong negative selection, as it has been observed with pathogens [61]. Interestingly enough, one of the overrepresented species belongs to the genus *Paenibacillus*. This genus contains either plant symbionts, counteracting insect herbivory, or known insect pathogens [80]. As its high prevalence might suggest that the larvae cannot filter out this bacterial species, it could be an interesting target for future studies in tephritid pest control.

### Is there a microbiome profile associated with cucurbit specialisation?

Given that previous studies have shown that dietary specialisation to particular host plants tends to be associated with specific microbiome profiles [10,11], we expected that the microbiomes of specialised cucurbit feeders would show a shared set of bacterial species, in particular species with known metabolisms relevant to cucurbit specialisation. Accordingly, the microbiomes of the target cucurbit feeders shared ASVs with signatures of selection (S4 Table). Several of these ASVs were Enterobacteriaceae, a bacterial family in which some taxa seem capable of degrading cucurbitacins [81], a class of toxins prevalent in cucurbits. Enterobacteriaceae are also prevalent among many tephritid species, including polyphagous species that do not infest cucurbits [82]. While this suggests that these bacteria might not play a specific role in cucurbit adaptation, we cannot exclude the possibility that cucurbit-feeding flies might harbor different Enterobacteriaceae with cucurbitacin degrading capacities that may be absent in non-cucurbit feeding tephritids. Other genera with ASVs with selection signatures included *Sphingobacterium*, *Pseudomonas* and *Acinetobacter*, which were associated with *Z*. *cucurbitae* [21]. Previous studies also suggested two other genera that might be important in cucurbit feeding: *Stenotrophomonas* and *Ochrobactrum* [21,35]. Although we did not find any ASVs belonging to these genera among all tested cucurbit feeders, different ASVs belonging to these genera had signatures of selection in different host fly species, indicating that they may play an important role in cucurbit feeding.

## Supporting information

S1 File Mutually Agreed Terms (MATs) on the use of genetic resources established between SUA and RMCA. (PDF)

S1 Table An overview of the sampling design. A) The general distribution of all samples collected in this study across all sites. This set was used for the differential abundance analysis, the null model analysis and neutral model analysis. B) a balanced subset of the original sampling design used in the analysis of α-diversity and β-diversity. Tephritid species are given as Db: *D*. *bivittatus*, Dc: *D*. *ciliatus*, Zc: *Z*. *cucurbitae*. Fruit host species are given as Cp: *Cucurbita pepo*, Cl: *Citrillus lanatus*, Cs: *Cucumis sativus*.
(XLSX)

S2 Table PERMANOVA on Unweighted Unifrac and Generalized Unifrac distances of third instar larval microbiomes. Calculated from from different fly species (*Dc*: *Dacus ciliatus*, *Db*: *D*. *bivittatus*, *Zc*: *Zeugodacus cucurbitae*) feeding on different host plants (*Cp*: *Cucurbita pepo*, *Cl*: *Citrullus lanatus*). The host plant *Cs*: *Cucumis sativus* is only included in the a posteriori comparison due to the lack of *Dc* found on *Cs*. Sheet “Main Test” contains the main PERMANOVA test while the *a posteriori comparison* can be found in sheet “Post hoc test”. n.s.: Not significant; *: P < 0.05, **: P < 0.01, ***: P < 0.001.
(XLSX)

S3 Table Neutral partitioning analysis from the Sloan neutral model for each fly species (*D*. *bivittatus*, *D*. *ciliatus*, *Z*. *cucurbitae*). UW stands for unweighted partitioning and AW stands for abundance-weighted partitioning. The neutral columns show the percentage of ASVs in the microbiome with an mean abundance-prevalence ratio that fits the expectation of the neutral assembly model (within 95% confidence interval). The below columns show the percentage of ASVs that have a lower prevalence than expected for their mean abundance predicted by the neutral model. The above columns show the percentages of ASVs that have a higher prevalence than expected for their mean abundance predicted by the neutral model. m gives the mean migration rate of all ASVs associated with a fly species.
(XLSX)

S4 Table Identification of Bacterial ASVs that fit with the neutral model expectation or that deviate from the neutral model expectation. The sheets SNM_DB, SNM_DC and SNM_ZC show the results of the identification for *D*. *bivitattus*, *D*. *ciliatus* and *Z*. *cucurbitae* respectively. The sheet Shared_Negative shows the ASVs that are less prevalent than expected in every cucurbit feeder (Below) while the sheet Shared_Positive shows the ASVs that are more prevalent than expected in every cucurbit feeder (Above). p = observed mean relative abundance, freq = observed prevalence of ASV, freq.pred = predicted prevalence of ASV, pred.lwr and pred.upr show the lower and upper confidence interval. bino.pred, bino.lwr, bino.upr, pois.pred and pois.lwr provide the predicted frequency, lower confidence interval and upper confidence interval for the binomial and Poisson model respectively. Type gives the identification of the ASV as to whether it fits the neutral expectation (Neutral) or is Above or Below the confidence interval.
(XLSX)

S1 Fig Principal Coordinates Analysis (PCoA) of microbial communities of third instar larvae of different fly species (*D*. *ciliatus*, *D*. *bivittatus* and *Z*. *cucurbitae*) feeding on different host plants (*C*. *pepo*, *C*. *lanatus*) as calculated from (a) Generalized Unifrac and (b) Unweighted Unifrac distances. Plots of the different fly species constitute partitions of the same plot.
(TIF)
