# Supplementary figures and images for: Deterministic and stochastic effects drive the gut microbial diversity in cucurbit-feeding fruit flies (Diptera, Tephritidae)
Source: PLoS One. 2025 Jan 24;20(1):e0313447. doi: 10.1371/journal.pone.0313447 (PMC11759365; doi:10.1371/journal.pone.0313447)

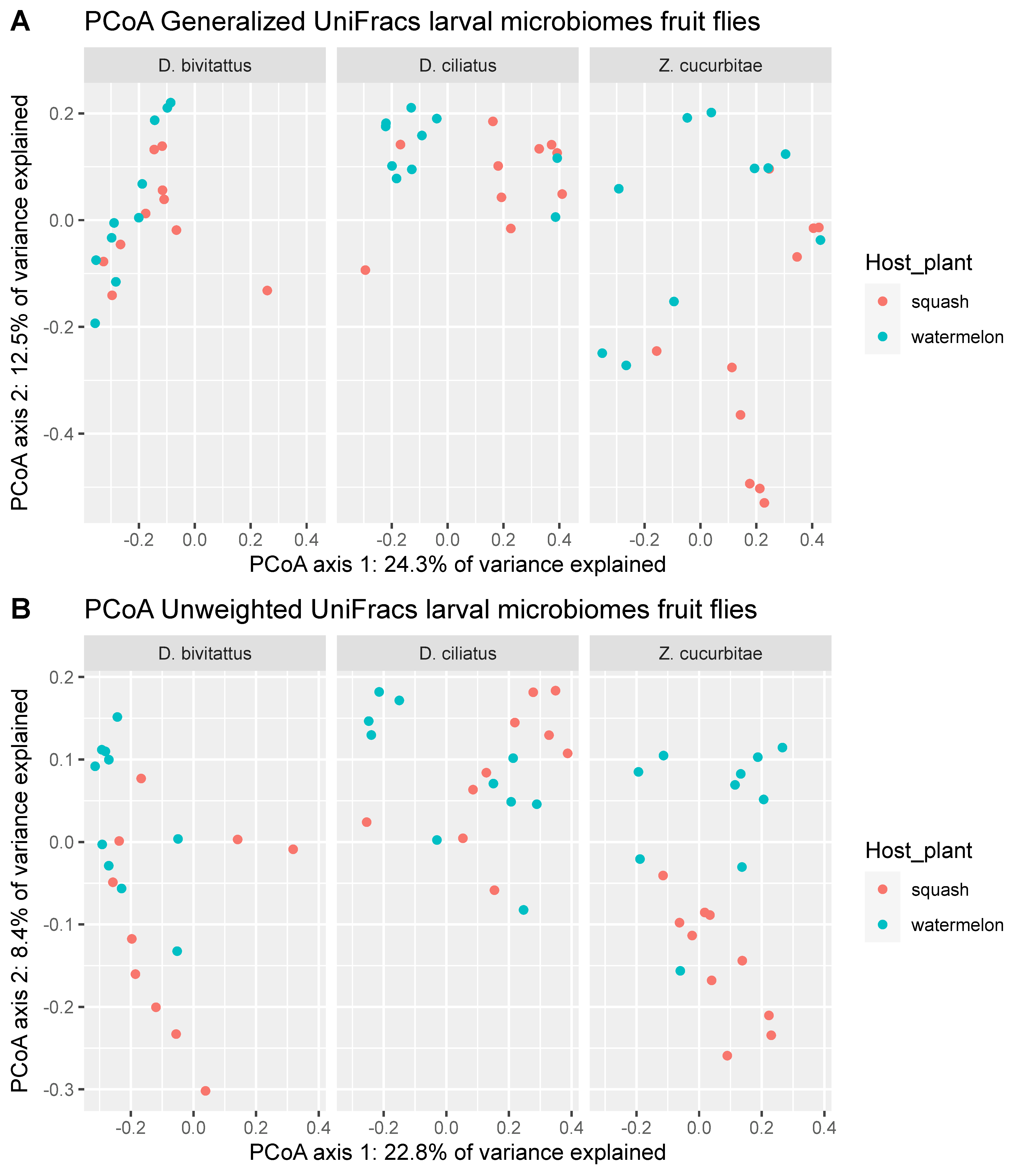

Supplement: S1 Fig — Plots of the different fly species constitute partitions of the same plot. (TIF) [file pone.0313447.s006.tif]
